# Arsenic in Paper

**Published:** 1885-01

**Authors:** 


### ARSENIC IN PAPER.

An article on "Arsenic as a Domestic Poison," by Dr. Edward S. Wood, in the fifth annual report of the Massachusetts State Board of Health, Lunacy and Charity, charges many cases of disease upon manufacturers of various colored papers. Wall paper is the most common source of domestic arsenical poisoning; green papers are not the only ones, but arsenic is found more commonly in wall paper of other colors. The cost of the paper is not a test, for expensive papers often contain it, while cheap ones may not. The most danger us of other papers are the glazed and plated, so largely used by children in Kindergarten schools. Some of these papers contain large quantities of arsenic, and are particularly dangerous, because children are inclined to put things in their mouths. Similar colored papers are used for covering boxes which often contain confectionery, and for wrapping it The green paper used for sassafras lozenges and the dark red or magenta colored paper used for checkerberry lozenges are highly arsenical. These poisonous papers are also used in the manufacture of theater tickets, playing cards, and everything else that is made from card board. Green color is not to be relied upon as principally poisonous, for red, brown and blue are specially arsenical. Arsenic is used in linen glaze and paper collars and cuffs. German fly paper is made by soaking coarse bibulous paper in a strong solution of arsenite of sodium and allowing it to dry. The following articles contain arsenical pigments, dies or mordants within the knowledge of the British National Health Society: Paper, fancy and surface colored, for covering card board boxes, for labels of all kinds, for advertising of all kinds, for advertising cards, for playing cards, for wrappers and cases for sweetmeats, for the ornamentation of children's toys, for covering books, for lamp shades, for paper hangings, for artificial leaves and flowers, lithographic color printing, book cloth and fancy bindings. Arsenical poisoning is produced by contact with objects containing arsenic, by inhaling the poisonous dust and gas that come from them, and by swallowing the material. Dr. Wood writes at length, and with much evident thought and care on the local and general symptoms of this poisoning, and gives a long list of illustrative cases that prove beyond question that arsenical papers cause serious acute complaints, and, indeed, many instances of death.

